# Alanine with the Precipitate of Tomato Juice Administered to Rats Enhances the Reduction in Blood Ethanol Levels

**DOI:** 10.1155/2015/280781

**Published:** 2015-12-02

**Authors:** Shunji Oshima, Sachie Shiiya, Yoshimi Tokumaru, Tomomasa Kanda

**Affiliations:** Research & Development Laboratories for Innovation, Asahi Group Holdings, Ltd., Ibaraki 302-0106, Japan

## Abstract

Delay in gastric emptying (GE) lowers the blood ethanol concentration (BEC) after alcohol administration. We previously demonstrated that water-insoluble fractions, mainly comprising dietary fiber derived from many types of botanical foods, possessed the ability to absorb ethanol-containing aqueous solutions. Furthermore, there was a significant correlation between the absorption of ethanol and lowering of BEC because of delay in GE. Here we identified dietary nutrients that synergize with the water-insoluble fraction of tomatoes to lower BEC in rats. Consequently, unlike tomato juice without alanine, tomato juice with 5.0% alanine decreased BEC depending on the delay in GE and mediated the ethanol-induced decrease in the spontaneous motor activity (an indicator of drunkenness). Our findings indicate that the synergism between tomato juice and alanine to reduce the absorption of ethanol was attributable to the effect of alanine on precipitates such as the water-insoluble fraction of tomatoes.

## 1. Introduction

Alcoholic beverages comprise the most commonly consumed foods by adults worldwide, and their inappropriate and excessive use is associated with an increased risk of several diseases [1]. Because alcohol is usually consumed with meals, understanding the effects of the diet on the pharmacokinetics of alcohol is important to reduce the harmful effects of alcohol on human health. Several dietary components are often employed to increase alcohol metabolism. It is well known that the oral intake of fructose or sucrose stimulates the elimination of alcohol from the bloodstream of healthy or alcoholic subjects [2–4]. Moreover, the ability of alanine to lower the blood ethanol concentration (BEC) may be attributed to the formation of pyruvate by oxidative deamination. Metabolic pathways generate NAD+, which facilitates alcohol oxidation in the liver via the conversion of pyruvate to lactate [5]. However, the most significant effect that foods can have on the absorption of alcohol is delaying the gastric emptying (GE). GE delivers nutrients to the duodenum after meals [6], and numerous reports show that delayed GE decreases BEC [7–12]. Alcohol consumed with a meal is likely released into the duodenum in a gradual manner, regardless of the composition of nutritional constituents, thus reducing BEC because of the reduction in the absorption rate [13]. Little is known about the effects of dietary components on the regulation of the rate of GE and its effect on the dynamics of alcohol metabolism.

We previously found that the water-insoluble fractions (WIFs) of several types of botanical foods besides tomatoes, mainly comprising water-insoluble dietary fibers, absorb ethanol-containing solutions. Moreover, the absorption of ethanol correlates with the inhibition of the blood ethanol elevation by delaying GE [14]. To the best of our knowledge, few studies have assessed the effects of dietary fibers on the dynamics of alcohol metabolism. In the current study, we analyzed BEC and the spontaneous motor activity as the indicators of drunkenness or intoxication. We were specifically interested in identifying nutrients that synergize with tomatoes to more effectively lower BEC.

## 2. Materials and Methods

### 2.1. Materials

Ethanol (99.5%) was purchased from Kanto Chemical Co., Inc. (Tokyo, Japan). Both the commercially available tomato paste and DL-alanine were kindly provided by Kagome Co., Ltd. (Tokyo, Japan) and Ajinomoto Healthy Supply, Inc. (Tokyo, Japan).

### 2.2. Sample Preparation

Tomato juice (5.8 Brix) was diluted 5-fold (w/v) using tomato paste and mineral water, and 200 mL of tomato juice was centrifuged at 3.000 rpm for 10 min. The supernatant, that is, the water-soluble fraction (WSF), and the precipitate, that is, the water-insoluble fraction (WIF), were diluted using mineral water to 200 mL. This yielded 200 mL of a WSF solution and 200 mL of a WIF suspension of tomato juice. Alanine (5.0 g) was added to 100 mL of each of the following: water, tomato juice, WSF, and WIF. Samples were stored at 5°C. The natural content of alanine in tomatoes is negligible (<0.01%) [15].

### 2.3. Preparation of Dry Powder of WIF Using Tomato Paste

Tomato paste (100 g) was added to 1 L of water, stirred thoroughly, and centrifuged at 3.000 rpm for 10 min. The supernatant was carefully removed. The washes were repeated until the Brix value of the supernatant solution reached 0. The precipitates were then frozen and freeze-dried to obtain WIF as a dry powder, which was stored at room temperature in a continuously evacuated desiccator until its ethanol-maintaining ability was assessed.

### 2.4. Determination of Ethanol-Maintaining Ability

Briefly, we prepared 15% (v/v) aqueous solutions of ethanol solution or suspensions containing 5.0% (w/v) alanine, 0.5% (w/v) dry WIF, or a mixture of 0.5% (w/v) dry WIF and 5.0% (w/v) alanine. A circular filter paper (110 mm diameter, Toyo Roshi Kaisha Ltd., Tokyo, Japan) was placed on a funnel, and 10 mL of each sample (or the 15% ethanol aqueous solution control) was poured on the filter paper. After 5 min, the filtered volume (mL) of ethanol solution was analyzed three times.

### 2.5. Animal Studies

All animal studies were conducted in accordance with the ethical guidelines for animal care, and the animal care committee of Asahi Group Holdings Ltd. (Ibaraki, Japan) approved the experimental protocol. F344 male rats (5 weeks old) were obtained from Japan SLC Inc. (Hamamatsu, Japan). The animals were housed in individual stainless steel cages in a room maintained at 25°C with 55% relative humidity and were allowed ad libitum access to laboratory chow pellets and tap water until use. For Experiment 1, the spontaneous motor activity of each rat weighing <360 g was measured continuously in a cage using the Supermex System (Muromachi Kikai, Japan), as described in previous studies [16, 17], for consecutive 4 h periods. Drunkenness induced by alcohol in rats decreases the spontaneous motor activity [18]. Four rats in each of the five groups (defined below) were fasted for 7 h. Before measuring the activity, the rats were orally given 20 mL/kg of tomato juice (tomato group), 5.0% aqueous alanine (alanine group), tomato juice with 5.0% alanine (tomato + alanine group), or water (water and control group). All groups were then given 8.45 mL/kg of 30% ethanol solution twice at 30 and 60 min intervals (total ethanol dose, 4.0 g/kg). The water group was given 8.45 mL/kg of water instead of ethanol solution. After the oral administration of ethanol, food and water were replenished and each rat was placed in a transparent plastic cage located in a sound-attenuating chamber. The sensor box, which was mounted in the center of the ceiling of the sound-attenuating chamber, detected the spontaneous motor activity; the signals were transmitted by an interface device to a computer and converted to the number of movements. Next, preliminary experiments were conducted to decide the time of blood collection after 2.0 g or 4.0 g/kg ethanol administrations to rats prior to Experiments 2 and 3. For Experiments 2A-2B, four rats weighing <270 g per group were fasted overnight and assigned to each group as follows: Before ethanol administration, the rats were orally given 20 mL/kg of tomato juice, 5.0% alanine aqueous solution, tomato juice with 5.0% alanine, or water (control) in Experiment 2A and an additional 20 mL/kg of tomato juice supernatant with or without 5.0% alanine or tomato juice precipitate with or without 5.0% alanine was given in Experiment 2B. The rats were then given 8.45 mL/kg of 30% ethanol solution twice, at 30 and 60 min intervals (total ethanol dose, 4.0 g/kg). Blood samples were then collected from the tail vein of each rat after 4 h because in preliminary experiments, using this method, we established that BEC peaked approximately 4 h after the administration of ethanol (4.0 g/kg) (see Figure 2). In Experiment 3, rats (*n* = 3 per group) weighing 240–340 g were fasted for 24 h and then orally given 20 mL/kg of tomato juice precipitate, 5.0% alanine aqueous solution, tomato juice precipitate with 5.0% alanine, or water (control). After 2.0 g/kg (8.45 mL/kg) of ethanol administration, blood samples were collected from the large vein of each rat after 2 h, when BEC reached maximal level (see Figure 2). After anesthetizing the rats with carbon dioxide, the stomach was ligated immediately at the cardiac and pyloric regions and harvested. The fluid in the stomach was diluted using distilled water to 50 mL. Residual ethanol in the stomach was calculated as a percentage of the total ethanol dose. Experiment 3 was performed twice to confirm repeatability of the result.

In Experiment 4, rats (*n* = 6 per group) weighing 370–480 g were fasted overnight and then orally given 20 mL/kg of tomato juice with 5.0% alanine or water (control). After 30 min, the rats were then orally or intraperitoneally given 16.9 mL/kg of 7.5% ethanol containing 0.9% NaCl solution (ethanol dose, 1.0 g/kg). After ethanol administration, blood samples were collected from the tail vein of each rat after 1 h.

### 2.6. Determination of BEC and Residual Ethanol in the Stomach

The amount of ethanol in the blood and residual ethanol in the stomach was determined using headspace gas chromatography, as previously described [19]. Briefly, blood or stomach-fluid samples (50 *μ*L) were treated immediately with 2.5 mL of ice-cold 4% (w/v) perchloric acid (PCA) with ethanol-d6 (Merck, 99% D anhydrous) added as an internal standard to a capped tube. The supernatant (2.0 mL) collected after centrifugation at 3.000 rpm for 10 min at 4°C was transferred to a glass vial containing 0.50 g of solid NaCl, and the PCA-treated samples were applied to a GC-MS system. GC-MS analysis was performed using an Agilent 6890 N gas chromatograph coupled to an Agilent MS-5975C inert XL mass-selective detector and an Agilent headspace sampler G1888 (Agilent Technologies, Little Fall, NY, USA). A capillary DB-Wax column (60 m × 0.25 mm, Agilent J & W GC Columns) was used to separate the peak ethanol fraction. The initial temperature of 35°C was maintained for 3 min and then increased to 80°C at 4°C/min; subsequently, the temperature was increased to 240°C at 20°C/min and then maintained at 240°C for 4 min. The split ratio was 1 : 20, and helium was used as a carrier gas and delivered at 1.0 mL/min. The injector and detector temperatures were 210°C and 250°C, respectively. The peaks of ethanol and ethanol-d6 in the treated samples were identified by comparing with the retention times and mass spectra of standards.

### 2.7. Statistical Analyses

All statistical analyses were performed using Dr. SPSS II software (SPSS Inc.). The difference in the spontaneous motor activity, BEC, or residual ethanol in the stomach among the groups was analyzed using one-way analysis of variance followed by Tukey's honest significant difference test in Experiments 1–3. Mean values with the same superscript letter are significantly different (*p* < 0.05). Filtered volumes at different time intervals (control versus alanine and WIF versus WIF + alanine) were compared using Student's *t*-test. The acceptable level of significance was 5% for each analysis. Simple linear regression (Pearson's) analysis was performed to examine the correlation between BEC (mg/mL) and the residual amount of ethanol in the stomach as well as to assess the relationship between the lowering of blood alcohol and GE. The relationship is expressed as the correlation coefficient (*r*). In Experiment 4, BEC between the control and tomato + alanine groups was compared using Student's *t*-test.

## 3. Results

**Figure 1:**
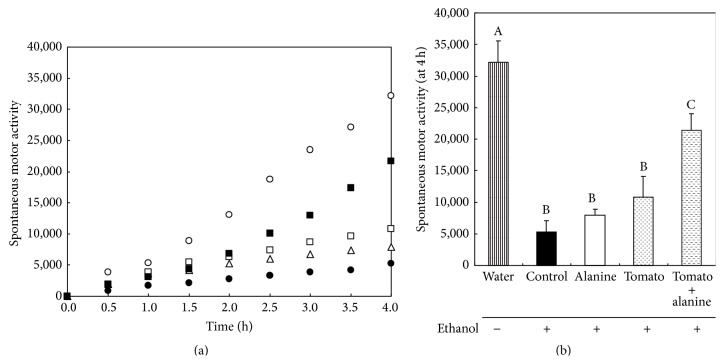
(a) The effects of a combination of tomato juice and alanine on the spontaneous motor activity 4 h following the ingestion of 4.0 g/kg of ethanol (Experiment 1). The rats were orally given 20 mL/kg of tomato juice (tomato group), 5.0% aqueous alanine (alanine group), tomato juice with 5.0% alanine (tomato + alanine group), or water (water and control group). Results are expressed as the mean values. ○, water (ethanol-free); ●, control; △, alanine; □, tomato; ■, tomato + alanine. (b) The spontaneous motor activity at 4 h following the ingestion of 4.0 g/kg of ethanol. Results are expressed as the mean and standard deviations (*n* = 4). Mean values with different uppercase letters differ significantly (ANOVA, post hoc Tukey's HSD test, *p* < 0.05) among five groups.

Spontaneous motor activity after the oral administration of a large amount of ethanol (4.0 g/kg) following the administration of each sample for 4 h periods is shown in Figures 1(a) and 1(b). The activity of the control group was significantly decreased compared with that of the water (ethanol-free) group. The activities of the tomato and alanine groups did not differ significantly from those of the control group. The activity of the tomato + alanine group was significantly greater than that of control and significantly lower than that of the water group.

**Figure 2:**
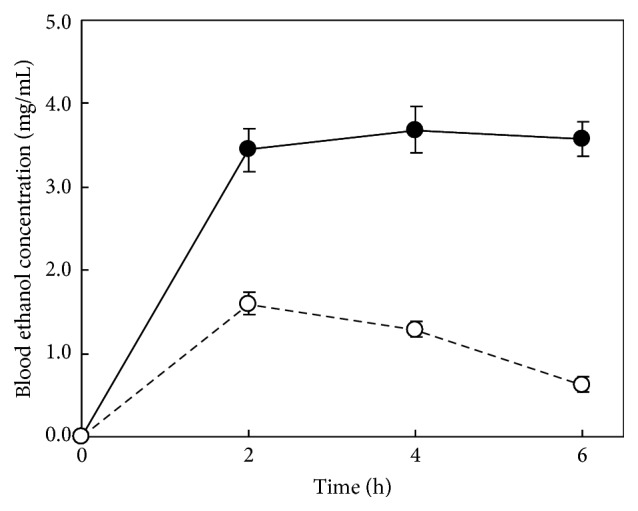
Changes in the blood ethanol concentration following the ingestion of 2.0 or 4.0 g/kg ethanol in preliminary experiments. Results are expressed as means and standard deviations. - - -○- - -, 2.0 g/kg (*n* = 10); —●—, 4.0 g/kg (*n* = 8).

**Figure 3:**
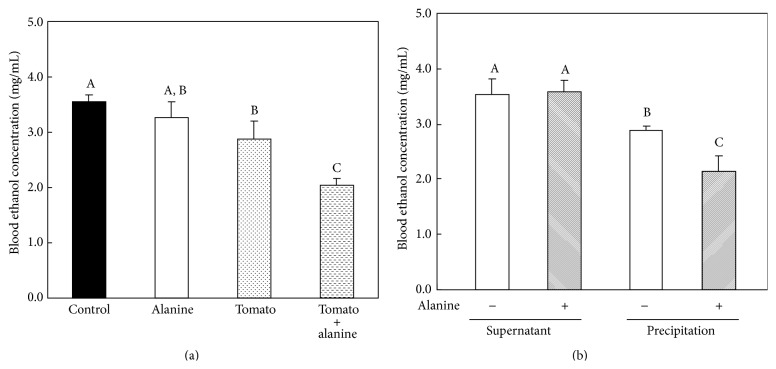
Blood ethanol concentrations 4 h following ingestion of 4.0 g/kg of ethanol after the administration of each sample in Experiments 2A and 2B. The rats were orally given 20 mL/kg of tomato juice, 5.0% alanine aqueous solution, tomato juice with 5.0% alanine, or water (control) in Experiment 2A and the tomato juice supernatant with or without 5.0% alanine or the tomato juice precipitate with or without 5.0% alanine in Experiment 2B. Results are expressed as mean and standard deviation (*n* = 4). Mean values with different uppercase letters differ significantly (ANOVA post hoc Tukey's HSD test, *p* < 0.05) among four groups.

We determined that BEC peaked approximately 4 h after the administration of 4.0 g/kg of ethanol in the preliminary experiment (Figure 2). BEC at 4 h after the oral administration of 4.0 g/kg of ethanol is shown in Figures 3(a) and 3(b). The BEC of the alanine group reached 3.26 mg/mL, similar to that of control group (3.54 mg/mL). The BEC (2.89 mg/mL) of the tomato group was significantly lower than that of the control group. Moreover, the BEC (2.04 mg/mL) of the tomato + alanine group was significantly lower than that of the three groups, including the tomato group. Next, we evaluated the supernatant or precipitate of tomato preparations with or without alanine. No difference in the BEC of the supernatant group with or without alanine (3.53 mg/mL versus 3.58 mg/mL) was detected; however, the BEC (2.89 mg/mL) of the precipitate group was significantly lower than that of the supernatant group. Furthermore, BEC after the administration of precipitate supplemented with alanine was significantly lower than that after the administration of precipitate without alanine (2.13 mg/mL versus 2.89 mg/mL).

**Figure 4:**
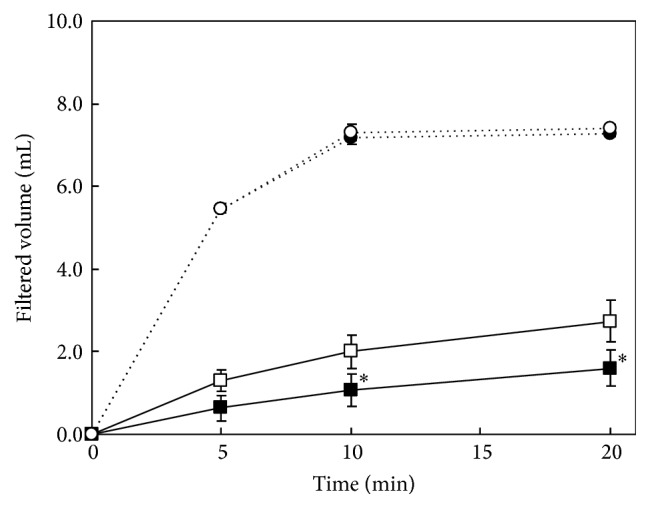
The ability of each sample to maintain a 15% (v/v) aqueous ethanol solution. Results are expressed as mean and standard deviation (*n* = 3). Asterisks indicate significant differences from the water-insoluble fraction (WIF) suspension group; *p* < 0.05, Student's *t*-test. - - -●- - -, control group; - - -○- - -, 5.0% aqueous alanine solution group; —□—, 0.5% WIF suspension group; —■—, 0.5% WIF + 5.0% alanine suspension group.

The ability of each sample to maintain 15% (v/v) ethanol is shown in Figure 4. During filtration, the aqueous ethanol solution in the fluids dripped through the filter paper more slowly when the samples strongly absorbed ethanol. The total filtered volume of the ethanol solution after 20 min (control) was 7.3 mL. The volume of the ethanol solutions containing 5.0% (w/v) alanine was equivalent to that of the control solution. The volume of the ethanol solution containing WIF was significantly lower than that of the control or alanine solutions. Moreover, the volume of WIF with alanine was significantly lower than that of WIF at 10 min and 20 min.

**Figure 5:**
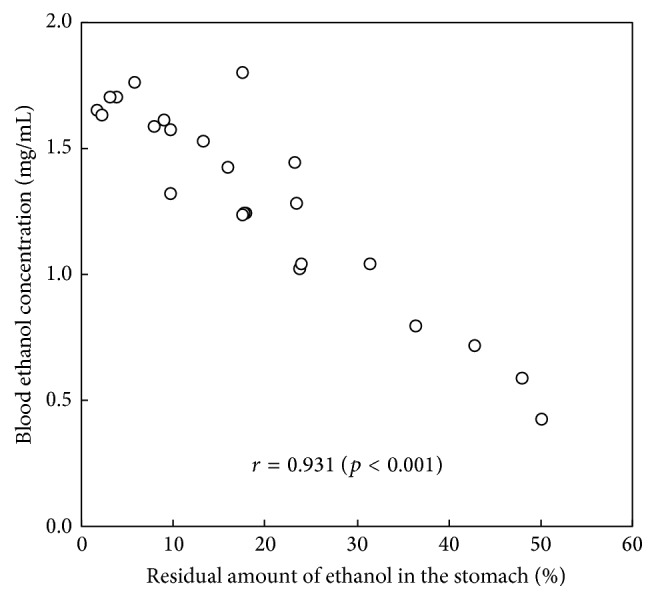
The correlation between the blood ethanol concentration and residual amount of ethanol in the stomach 2 h after ingestion of 2.0 g/kg ethanol after the administration of each sample in Experiment 3. Results represent the values of each rat (*n* = 24) and the correlation coefficient (*r*).

BEC and residual ethanol concentration in the stomach 2 h after the administration of 2.0 g/kg ethanol are presented in Table 1. The BECs of the control and the alanine groups 2 h after the administration of 2.0 g/kg of ethanol were 1.52 or 1.68 mg/mL and 1.47 or 1.66 mg/mL, respectively. In contrast, the BEC of the tomato juice precipitate with alanine group (0.57 or 0.95 mg/mL) was significantly lower than that of the tomato juice precipitate group (1.17 or 1.41 mg/mL). The residual gastric content of ethanol in the water control, alanine, tomato juice precipitate, and tomato juice precipitate with alanine groups was 16.9% or 6.5%, 15.3% or 2.4%, 22.4% or 13.9%, and 47.1% or 28.1%, respectively. There was no significant difference between the control and alanine groups; however, the amount of gastric ethanol was increased significantly in the tomato juice precipitate with alanine group compared with that of the tomato juice precipitate group. A simple linear regression (Pearson's) analysis was performed to examine the correlation between the residual amount of ethanol in the stomach and the BEC of each rat in the four groups. The correlation coefficient (*r*) for the difference between the residual amount and BEC was 0.931, and the two parameters correlated significantly (*p* < 0.001, Figure 5).

**Figure 6:**
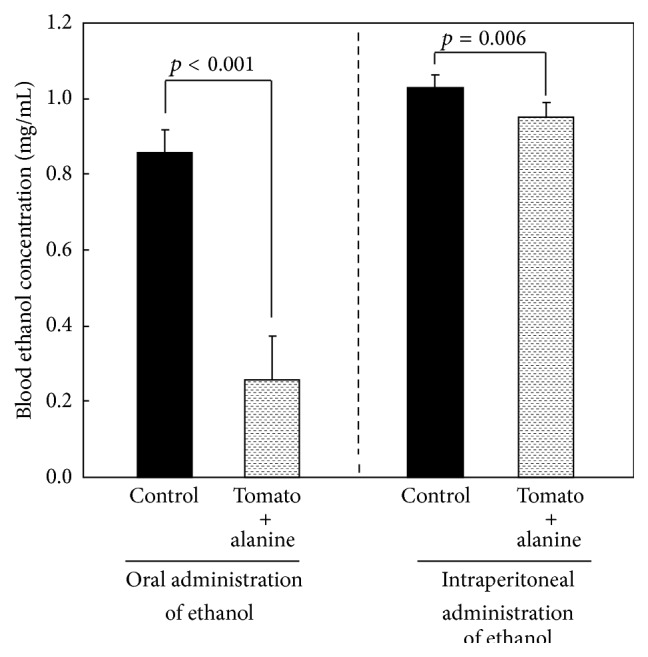
Blood ethanol concentrations 1 h after oral or intraperitoneal administration of 1.0 g/kg of ethanol (Experiment 4). Thirty minutes later, the rats received 20 mL/kg of either tomato juice with 5.0% alanine or water (control) orally. Results are expressed as mean and standard deviation (*n* = 6). Blood ethanol concentrations between control and tomato + alanine group were compared using the Student *t*-test.

BEC at 1 h after the oral or intraperitoneal administration of 1.0 g/kg of ethanol is shown in Figure 6. When rats were orally given ethanol, the BEC of the tomato juice with 5.0% alanine group (0.26 mg/mL) was significantly lower than that of the control group (0.86 mg/mL). When rats were intraperitoneally given ethanol, BEC of the tomato juice with 5.0% alanine (0.95 mg/mL) group was significantly lower than that of the control group (1.03 mg/mL); however, the lowering was weaker than that observed after the oral administration of ethanol.

## 4. Discussion

Tomatoes contain various nutrients and functional components, including carotenoids, polyphenols, and phytosterols [20, 21]. In this study we found that the water-soluble fraction of tomato juice contains 4.5% sugar (glucose and fructose) and had no effect on BEC, whereas WIF lowered BEC. A 0.7% suspension of WIF solids in water corresponded to 0.14 g/kg, most of which was dietary fiber (70.7%) followed by small amounts of lipids or water-insoluble proteins. It seems unlikely that WIF accelerated alcohol metabolism; thus, we suspect that WIF inhibited alcohol absorption in the gastrointestinal tract. Ushida et al. previously reported that the administration of a concentrated tomato supernatant attenuates elevated BEC [22]. However, the enriched water-soluble components were present in a concentrated tomato supernatant solution containing 11.3% glucose and 10.7% fructose. In contrast, tomatoes contain approximately 1%-2% glucose and fructose [23, 24]. Oral fructose (1.0 g/kg) significantly lowers the peak BEC in humans [4]. Thus, higher quantities of sugars in the supernatant may contribute to the lowering of BEC. In contrast, Stice et al. found that whole tomato powder reduces the severity of alcohol-induced steatosis or hepatic inflammatory foci and that a lipid-soluble tomato extract has no effect on these outcomes [25]. In this study, we did not identify the active components of whole tomatoes, and the lipid-soluble constituents were not measured. However, tomato varieties do contain dietary fiber [21]. Therefore, the results strongly suggest that the water-insoluble dietary fiber in whole tomatoes was responsible for reducing the adverse effects of alcohol by inhibiting alcohol absorption. To our knowledge, no previously published studies have addressed this question.

The initial goal of our study was to identify nutrients such as amino acids, sugars, and lipids that synergize with tomato WIF to produce effective functional foods for humans in the future. In an animal study, we evaluated the lowering of BEC because of supplementing tomato juice with one of the 22 amino acids. Supplementation of tomato juice with 3%–5% (w/v) of 7 amino acids (phenylalanine, histidine, ornithine, arginine, tryptophan, methionine, and alanine) significantly reduced BEC. Eighty percent of the free amino acids that naturally occur in tomatoes are glutamate, gamma-aminobutyrate, glutamine, and aspartate, and the alanine content of tomatoes is approximately < 0.01% [15], too low to influence BEC. We instead focused on the 7 amino acids that synergized with the components of tomatoes, in particular alanine, a superior tasting, sweet substance [26]. Oral administration of 4 g/kg alanine decreases the BEC of mice given 2 g/kg of ethanol [8]. However, an oral dose of 0.95 mmol (84.6 mg)/kg alanine does not affect rats given 1 g/kg ethanol [27]. In this study, the BEC of rats given 1.0 g/kg of alanine and 2.0 or 4.0 g/kg of ethanol did not significantly differ from those of the controls. In contrast, the BECs of rats given precipitate supplemented with alanine were significantly lower than that of the controls. We were surprised that the supernatant supplemented with alanine had no effect on BEC, but our data suggests that alanine enhanced the ethanol-maintaining activity of WIF, whereas alanine alone had no effect. Further studies will be required to determine the mechanism responsible for this effect. In any event, the addition of alanine to tomato juice significantly increased the gastric content of residual ethanol. This augmentation of the ethanol-maintaining activity caused by WIF supplemented with alanine is likely the result of increasing the residual gastric content of ethanol. In case of the intraperitoneal administration of ethanol, tomato with alanine significantly decreased BEC. This effect may imply the metabolic acceleration of ethanol in the liver caused by the small dose of ethanol. However, the effect was weaker than that on oral ethanol administration. It is an undoubted fact that BEC lowering effect not involving absorptive process of ethanol is limited. We believe that our data clearly demonstrates that the delay in the GE of ethanol decreased BEC and that gastric ethanol was strongly correlated with BEC. Furthermore, the addition of alanine to tomato juice precipitate significantly increased the residual gastric content of ethanol to 47.1% or 28.1%, which is equivalent to 604 or 432 mg/kg ethanol compared with control (16.9% or 6.5%). In the body, ingested alcohol is mainly dissolved in aqueous fluids because alcohol is insoluble in body fat. Thus, alcohol is suitable for use as a solute to measure the volume of total body water [28]. In this study, we did not determine rats body-water volume, but the body-water volume was previously reported to be approximately 66% [29]. Therefore, the difference in BEC corresponds to approximately 0.92 or 0.65 mg/mL (604/660 or 432/660). The measured BEC was 0.95 or 0.73 mg/mL (1.52−0.57 or 1.68−0.95), approximately equivalent to the calculated values. The rough consistency suggests that the quantity of unabsorbed or residual ethanol in the stomach is sufficient to explain the decrease in BEC. Further studies will be required to determine an effective dose of tomato components and alanine for humans who consume alcohol for the purpose of ameliorating drunkenness.

## 5. Conclusions

Alanine enhanced the capacity of tomato juice to delay GE, thus lowering BEC. The administration of tomato juice supplemented with alanine ameliorated the spontaneous motor activity in rats given a high dose of ethanol. Alanine potentiated the effect of WIF, which comprises mainly dietary fiber present in tomato juice, although alanine alone had no effect. Hence, we conclude that tomato juice precipitate and alanine synergized to decrease ethanol absorption.

## Figures and Tables

**Table 1:** Blood ethanol concentration and residual ethanol in the stomach 2 h after the administration of 2.0 g/kg of ethanol in Experiment 3.

	BEC	Residual amount of ethanol in stomach
	mg/mL	% dose
(First)		
Control	1.52 (0.25)^a^	16.9 (6.8)^a^
Alanine	1.47 (0.17)^a^	15.3 (7.4)^a^
Tomato juice precipitate	1.17 (0.12)^a^	22.4 (7.9)^a^
Tomato juice precipitate with alanine	0.57 (0.15)^b^	47.1 (3.7)^b^
(Second)		
Control	1.68 (0.10)^a^	6.5 (3.0)^a^
Alanine	1.66 (0.04)^a^	2.4 (0.7)^a^
Tomato juice precipitate	1.41 (0.18)^a^	13.9 (5.1)^a^
Tomato juice precipitate with alanine	0.95 (0.14)^b^	28.1 (7.2)^b^

Data are expressed as means and standard deviation (mean ± SD, *n* = 3). Means values with different lowercase letters are significantly different (ANOVA post hoc Tukey's HSD test, *p* < 0.05) among four groups. The secondary experiment was constructed to confirm repeatability of their effects.

## Abbreviations

BEC: Blood ethanol concentration
WSS: Water-soluble supernatant
WIP: Water-insoluble precipitation
GE: Gastric emptying.
